# Precision prevention of colorectal neoplasia in patients undergoing hemorrhoid surgery: an explainable machine learning model for identifying the risk of precancerous neoplastic polyps

**DOI:** 10.3389/fcell.2026.1834613

**Published:** 2026-05-28

**Authors:** Qifan Yang, Lu Nie, Ying Chu, Peipei Luo

**Affiliations:** 1 Department of Gastroenterology, Wujin Hospital Affiliated with Jiangsu University, Changzhou, China; 2 Department of Radiology, Changzhou Maternal and Child Healthcare Hospital, Changzhou, China; 3 Department of Laboratory Medicine, Wujin Hospital Affiliated with Jiangsu University, Changzhou, China

**Keywords:** hemorrhoid surgery, machine learning, precancerous neoplastic polyps, predictive model, risk factors

## Abstract

**Background:**

Hemorrhoids are common and frequently present with hematochezia. However, these symptoms can overlap with manifestations of precancerous neoplastic polyps (PNP) and early colorectal cancer. Although colonoscopy is recommended for patients with hematochezia, it is often not completed in routine practice for various reasons, which may lead to missed diagnoses in some patients. We built and validated a machine learning–based model to identify patients undergoing surgery for severe hemorrhoids who are at elevated risk of PNP, with the aim of supporting clinical decision-making and prevent colorectal neoplasms.

**Methodology:**

We enrolled 589 patients who underwent hemorrhoid surgery at Wujin People’s Hospital, between January 2021 and January 2025 and completed colonoscopy within 1 year before or after surgery. Demographic and clinical data recorded at initial admission comprised sex, age, body mass index (BMI), smoking, drinking, diabetes, and hypertension. Feature selection was conducted using least absolute shrinkage and selection operator (LASSO) regression, after which the dataset was randomly partitioned into training and testing cohorts at an 8:2 ratio. Our study constructed Seven machine learning models. The area under the receiver operating characteristic curve (AUROC) and standard classification metrics were used to assess discriminative performance; calibration was assessed with the Brier score. Shapley additive explanations (SHAP) -based attribution analysis was performed for the best-performing model. Finally, decision curve analysis (DCA) and risk stratification were used to assess clinical utility.

**Results:**

LASSO identified age, sex, BMI, smoking, drinking, and hypertension as key predictors. Among the evaluated models, RF achieved the highest training-set AUROC (0.892). In the testing set, the AUROC was 0.738 with a Brier score of 0.172, suggesting acceptable calibration and reasonable overall stability. Risk stratification according to tertiles of predicted probability demonstrated a distinct gradient in the prevalence of PNP, increasing from 7.5% in the low-risk group to 23.1% in the intermediate-risk group and 53.8% in the high-risk group. SHAP analysis showed that age contributed most to the predictions, followed by drinking, BMI, smoking, sex, and hypertension.

**Conclusion:**

This model may help clinicians more accurately identify high-risk individuals among patients undergoing hemorrhoid surgery, potentially reducing missed PNP and strengthening colorectal cancer prevention.

## 1 Introduction

Hemorrhoids are a common anorectal condition that typically presents with hematochezia, discomfort during defecation, or perianal discomfort (Lee, 2015; Hawkins et al., 2024). The diagnosis is primarily based on medical history, visual inspection of the anus, digital rectal examination, and, when necessary, anoscopy or proctoscopy. However, these symptoms are not specific to hemorrhoids and can also occur in patients with colorectal polyps or early-stage colorectal cancer, which complicates the clinical differential diagnosis (Goulston et al., 1986). Precancerous neoplastic polyps (PNP), including colorectal adenomatous polyps and serrated polyps (SP), are common in clinical practice and are important precursor lesions of colorectal cancer (Liang et al., 2020; Dornblaser et al., 2024). Studies have shown that colonoscopy-based screening with early detection and removal of PNP can significantly reduce colorectal cancer incidence and mortality (Bretthauer et al., 2022). Therefore, colonoscopy is strongly recommended for patients presenting with hematochezia (Goulston et al., 1986). The American Society of Colon and Rectal Surgeons (ASCRS) states that, when feasible, patients with hemorrhoidal bleeding should undergo colonoscopic evaluation; however, the supporting evidence remains insufficient (Hawkins et al., 2024). In our study, we observed that some patients in routine clinical practice are unable or unwilling to complete recommended evaluations because of financial constraints or poor adherence. This may lead to missed diagnoses of colorectal polyps or early-stage colorectal cancer in high-risk individuals. Notably, hemorrhoids are an important risk factor for colorectal polyps (Toyoshima et al., 2023). Moreover, risk factors for PNP substantially overlap with those for hemorrhoids (Yamaji et al., 2006; Lee et al., 2019; Hong et al., 2022). Therefore, identifying patients at high risk for PNP among those undergoing hemorrhoid surgery is necessary.

Most existing studies have examined hemorrhoids and PNP separately, whereas evidence on risk factors for concomitant PNP in patients undergoing hemorrhoid surgery remains limited (Hong et al., 2022; Aimaierjiang and Simayi, 2024). As machine learning approaches continue to advance in clinical research, they offer clear advantages for integrating multiple variables, modeling nonlinear relationships, and enabling individualized risk prediction (Silva et al., 2022). Compared with traditional statistical methods, machine learning may enhance diagnostic accuracy and efficiency by integrating clinical, imaging, and laboratory data, reinforce the evidence base for risk factor identification, and facilitate more precise treatment strategies (Silva et al., 2022; Deng et al., 2024a). At the same time, strategies such as explainable artificial intelligence, cross-cohort validation of risk scores, and the integration of radiomics with gene expression have been widely employed for prognostic prediction and risk stratification across various malignant tumors (Dogra and Hasija, 2025; Chen et al., 2025; Kawahara et al., 2025). Collectively, these findings suggest that artificial intelligence-based models may offer a valuable framework for identifying high-risk populations and facilitating individualized clinical decision-making. Accordingly, we enrolled patients undergoing hemorrhoid surgery and applied machine learning algorithms to identify factors associated with PNP and to develop a predictive model. We sought to delineate PNP-associated risk factors and to develop a predictive model. This work aimed to support early risk stratification to identify high-risk patients, prevent colorectal neoplasms, and generate evidence to inform clinical practice.

## 2 Materials and methods

### 2.1 Data collection

Our study enrolled patients treated at Wujin Hospital Affiliated with Jiangsu University between 2021 and 2025. Eligibility required hemorrhoid surgery for severe hemorrhoidal disease, defined as grade III–IV internal hemorrhoids according to the Goligher classification, and completion of colonoscopy within 1 year before or after the operation. The exclusion criterion was the absence of a pathological diagnosis after polyp detection for any reason. Patients were stratified into PNP and non-PNP groups using colonoscopy and histopathological findings as the reference standard. PNP was defined as histopathologically confirmed precancerous colorectal lesions, including adenomatous polyps and serrated lesions with malignant potential. Baseline variables at first admission comprised demographic characteristics (sex and age), metabolic comorbidities (diabetes and hypertension), smoking and drinking, and BMI. The outcome was the presence of PNP, defined by colonoscopy and histopathological findings. This study received approval from the Ethics Committee of Wujin Hospital Affiliated with Jiangsu University (2025-SR-327).

### 2.2 Statistical methods

Continuous variables were z-score standardized to reduce the influence of differences in measurement scale on model training. Continuous variables showed non-normally distribution and were therefore summarized as medians; comparisons between two independent groups were performed using the Mann–Whitney U test. Categorical variables are presented as counts (percentages) and were compared using the chi-square (χ2) test. Normality testing (Kolmogorov–Smirnov) indicated departures from a normal distribution for the quantitative variables. An optimal α was selected for least absolute shrinkage and selection operator (LASSO) regression to perform feature selection, thereby yielding a parsimonious model while limiting multicollinearity. The dataset was divided into training and testing subsets at an 8:2 ratio using stratified random sampling according to PNP status, in order to preserve the prevalence of PNP in both subsets. We fit each algorithm using the training data, including logistic regression (LR) and support vector machine (SVM), instance- and tree-based methods (KNN and DT), ensemble learners (RF and AdaBoost), and a neural network (NN). Hyperparameters were tuned via grid search, and five-fold cross-validation was used to evaluate model stability and generalizability. On the testing set, we evaluated AUROC and classification metrics (accuracy, precision, recall, and F1 score) and selected the final model according to its overall performance profile. In addition, Shapley additive explanations (SHAP) wes used to the predictions and visualizing the relative importance of key variables in model decision-making. Python 3.7 served as the platform for all statistical computations.

For clinical interpretability, predicted probabilities in the testing set were ranked and categorized into tertiles to define low-, intermediate-, and high-risk strata (cut points at the 33.3rd and 66.7th percentiles). In addition, decision curve analysis was performed to evaluate the net benefit of the final model across a range of threshold probabilities, compared with treat-all and treat-none strategies.

## 3 Results

### 3.1 Etiological investigation

**FIGURE 1:**
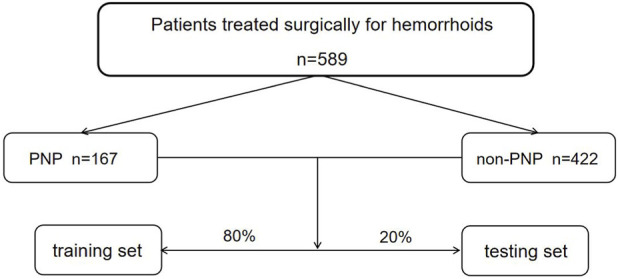
Flowchart of patient selection and dataset division.

**FIGURE 2:**
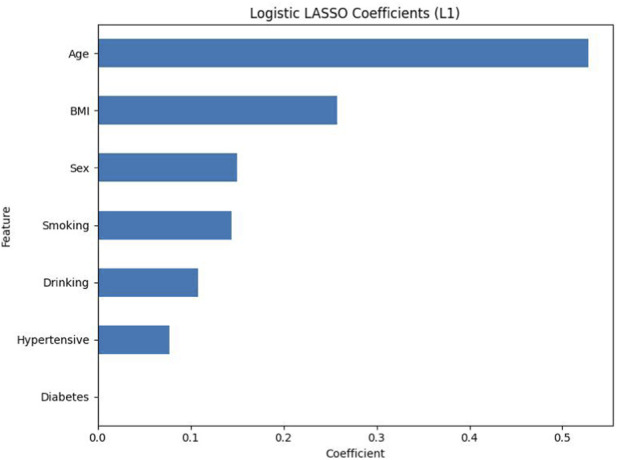
Feature selection using least absolute shrinkage and selection operator (LASSO) regression. Regression coefficients of candidate predictors obtained from LASSO logistic regression are shown. Non-zero coefficients indicate variables retained in the final model, whereas coefficients shrunk to zero were excluded. Larger coefficient magnitudes indicate stronger contributions to prediction. Age contributed most prominently, followed by BMI, sex, smoking, drinking, and hypertension; diabetes was eliminated during regularization.

This study included 589 patients treated surgically for hemorrhoids at Wujin Hospital Affiliated with Jiangsu University. Among them, 167 (28.7%) had PNP confirmed on histopathology after colonoscopy performed within 1 year before or after surgery (Figure 1). Table 1 summarizes baseline characteristics of patients undergoing hemorrhoid surgery. The PNP and non-PNP groups were largely comparable in terms of diabetes and hypertension status. However, patients in the PNP group were older, predominantly male, and had a higher BMI. Additionally, they were more likely to have a history of smoking and alcohol use. Cross-validation was conducted across a range of regularization parameters to determine the optimal value. To reduce reliance on univariate comparisons, all candidate variables were subsequently included in LASSO regression for feature selection prior to model development (Figure 2). LASSO regression identified key predictors, including age, BMI, smoking, drinking, and a history of hypertension, as being associated with PNP. These results are shown in a bar plot.

**TABLE 1:** Baseline characteristics and univariate analysis of hemorrhoid surgery patients.

Variables	Total (n = 589)	PNP (n = 167)	Non-PNP (n = 422)	Z/χ^2^	P value
Sex	​	​	​	11.006	​
Male	273 (46.3%)	96 (57.5%)	177 (41.9%)	​	<0.001
Female	316 (53.7%)	71 (42.5%)	245 (58.1%)	​	​
Age (years)	55 (45, 63)	61 (52, 66)	52 (44, 61)	5.925	<0.001
BMI (kg/m^2^)	23.99 (22.43, 25.09)	24.23 (22.68, 27.03)	23.71 (22.23, 24.98)	3.174	0.002
Diabetes	​	​	​	0.523	​
No	532 (90.3%)	148 (88.6%)	384 (91.0%)	​	0.470
Yes	57 (9.7%)	19 (11.4%)	38 (9.0%)	​	​
Hypertension	​	​	​	2.491	​
No	464 (78.8%)	124 (74.3%)	340 (80.6%)	​	0.115
Yes	125 (21.2%)	43 (25.7%)	82 (19.4%)	​	​
Smoking	​	​	​	11.294	​
No	470 (79.8%)	118 (70.7%)	352 (83.4%)	​	<0.001
Yes	119 (20.2%)	49 (29.3%)	70 (16.6%)	​	​
Drinking	​	​	​	7.302	​
No	393 (66.7%)	97 (58.1%)	296 (70.1%)	​	0.007
Yes	196 (33.3%)	70 (41.9%)	126 (29.9%)	​	​

PNP, precancerous neoplastic polyp.

### 3.2 Development and validation of predictive models

Grid search with cross-validation (GridSearchCV) was used to tune each machine learning model and evaluate performance within the training set. The area under the receiver operating characteristic curve (AUROC) served as the primary metric, supplemented by accuracy, recall, F1 score and precision for comprehensive assessment (Table 2). In addition, the Brier score was used to assess overall performance by jointly reflecting discrimination and calibration. Overall, the RF model showed the best discrimination.

**TABLE 2:** Performance indicators of the machine learning model in the training set.

Model	Accuracy	Precision	Recall	F1-score	AUC
RF	0.737	0.708	0.737	0.683	0.892
SVM	0.695	0.636	0.695	0.645	0.763
LR	0.729	0.691	0.729	0.668	0.693
NN	0.737	0.707	0.737	0.697	0.819
KNN	0.771	0.756	0.771	0.755	0.875
DT	0.695	0.627	0.695	0.636	0.837
AdaBoost	0.695	0.572	0.695	0.604	0.728

*RF*, random forest; *SVM*, support vector machine; *LR*, logistic regression; AdaBoost; *NN*, neural networks; *KNN*, K-Nearest Neighbors; *DT*, decision trees.

**FIGURE 3:**
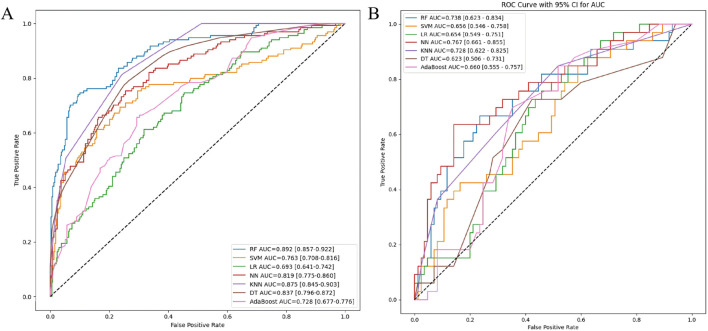
The area under the receiver operating characteristic curve (AUROC) of machine-learning models. **(A)** ROC curves of the machine-learning models in the training set. **(B)** ROC curves of the machine-learning models in the testing set. The AUROC values with 95% confidence intervals are shown for each model. The ROC curve plots sensitivity against 1−specificity, and the dashed diagonal line indicates no discrimination (AUC = 0.5). Abbreviations: RF, random forest; SVM, support vector machine; LR, logistic regression; NN, neural network; KNN, k-nearest neighbors; DT, decision tree; AdaBoost, adaptive boosting.

**FIGURE 4:**
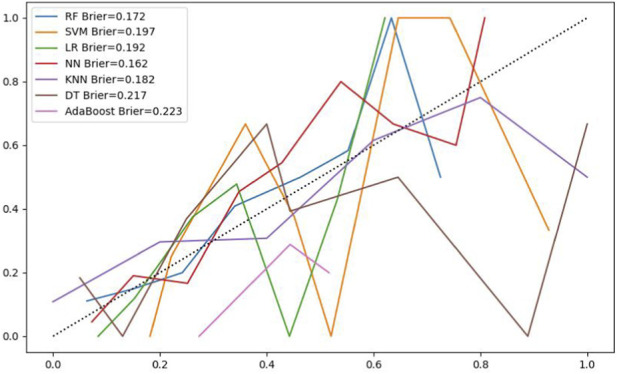
Calibration curves of machine-learning models. Calibration curves comparing predicted probabilities with observed event rates for the machine-learning models in the training set. The x-axis represents the predicted probability of precancerous neoplastic polyps, and the y-axis represents the observed probability. The diagonal dashed line indicates perfect calibration; curves closer to this line indicate better agreement between predictions and outcomes. Brier scores are shown in the legend, with lower values indicating better overall calibration. Abbreviations: RF, random forest; SVM, support vector machine; LR, logistic regression; NN, neural network; KNN, k-nearest neighbors; DT, decision tree; AdaBoost, adaptive boosting.

**FIGURE 5:**
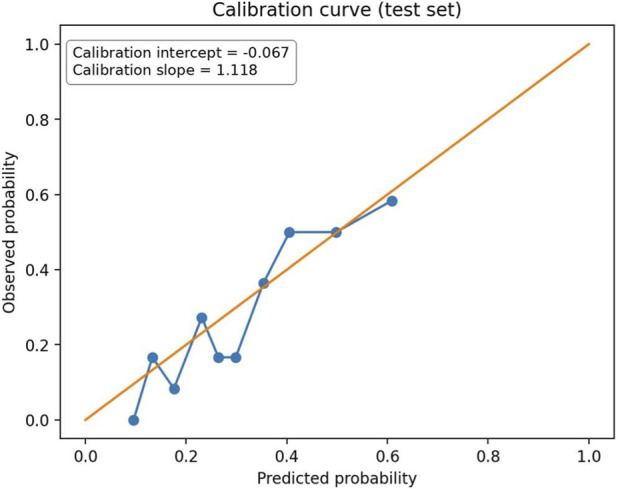
Calibration curve of the RF model in the testing set. Calibration curve comparing predicted probabilities with observed event rates for the random forest (RF) model in the testing set. The x-axis represents the predicted probability of precancerous neoplastic polyps, and the y-axis represents the observed probability. The diagonal dashed line indicates perfect calibration; curves closer to this line indicate better agreement between predictions and outcomes. Calibration assessment yielded an intercept of −0.067 and a slope of 1.118, suggesting acceptable agreement between predicted and observed risks. Abbreviations: RF, random forest; PNP, precancerous neoplastic polyps.

RF showed the strongest discrimination, with an AUROC of 0.892 in the training cohort (95% CI 0.857–0.922; Figure 3) and 0.738 on the testing set. For the other algorithms (training cohort), the AUROC and corresponding uncertainty ranges were: SVM, 0.763 [0.708, 0.816]; LR, 0.693 (0.641–0.742); NN, 0.819 with a 95% CI of 0.775–0.860; KNN, 0.875, CI = 0.845–0.903; DT, 0.837, with the interval spanning 0.796 to 0.872; and AdaBoost, 0.728, 95% confidence interval 0.677–0.776. In the testing set, discrimination decreased across models but showed a similar pattern, with AUROCs of 0.656 (SVM), 0.654 (LR), 0.767 (NN), 0.728 (KNN), 0.623 (DT), and 0.660 (AdaBoost). The RF model demonstrated acceptable calibration, with a Brier score of 0.172 (Figure 4), supporting overall stability. Although RF model retained acceptable discriminative performance in the testing set, its AUROC declined from 0.892 in the training set to 0.738 in the testing set. This reduction in performance indicates that the model may be affected by some degree of bias or overfitting. Therefore, further validation in independent external cohorts remains necessary. Calibration assessment in the testing set yielded a calibration intercept of −0.067 and a calibration slope of 1.118, suggesting overall acceptable agreement between predicted and observed risks (Figure 5).

**FIGURE 6:**
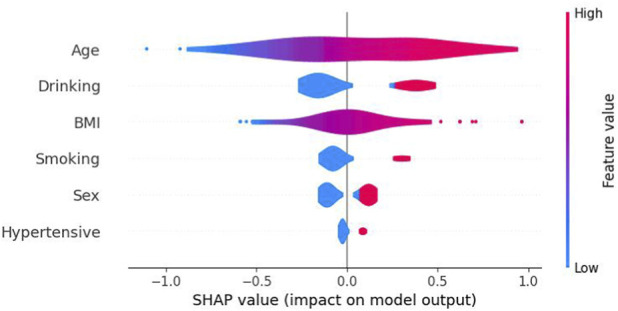
SHAP summary plot of the RF model for predicting PNP. Each point represents an individual sample. The x-axis indicates the SHAP value (impact on model output), and color denotes feature magnitude (red, high; blue, low). Features are ordered by importance based on mean absolute SHAP values.

The SHAP feature importance ranking showed that age contributed most to the model predictions, followed by drinking, BMI, smoking, and sex; hypertension had a smaller contribution (Figure 6).

### 3.3 Clinical utility and risk stratification

**FIGURE 7:**
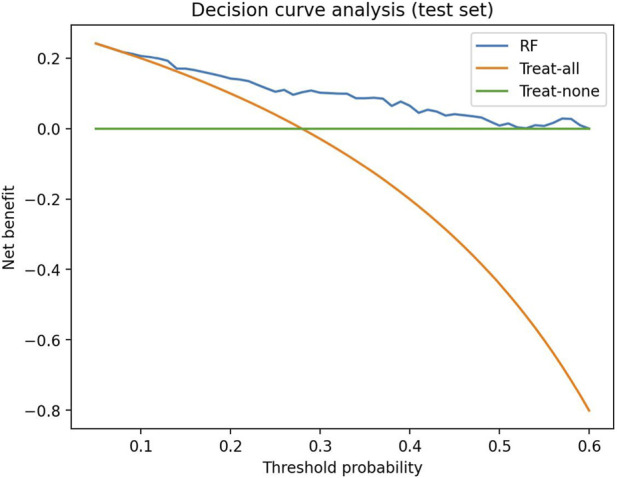
Decision curve analysis (DCA). DCA of the random forest (RF) model in the testing set. Net benefit is plotted across a range of threshold probabilities, comparing the RF model with treat-all and treat-none strategies.

Using the Youden index obtained from the training set, the optimal probability cutoff was identified as 0.367. When applied to the testing set, this threshold produced a sensitivity of 0.636 and a specificity of 0.800, with a positive predictive value of 0.553 and a negative predictive value of 0.850. The model achieved an overall accuracy of 0.754 and an F1 score of 0.592. To improve clinical applicability, patients in the testing set were subsequently categorized into low-, intermediate-, and high-risk groups according to tertiles of the predicted probabilities. The prevalence of PNP showed a progressive increase across these risk categories, rising from 7.5% (3/40) in the low-risk group and 23.1% (9/39) in the intermediate-risk group to 53.8% (21/39) in the high-risk group, suggesting a distinct risk gradient. Decision curve analysis (DCA) further showed that, across a clinically meaningful range of threshold probabilities, the RF model yielded greater net benefit than either the treat-all or treat-none strategy (Figure 7), supporting its potential value for prioritizing colonoscopy in patients undergoing hemorrhoid surgery (Table 3).

**TABLE 3:** Risk stratification according to predicted probabilities in the testing set.

Risk group	Predicted probability	n	PNP, n (%)
Low-risk	≤0.226	40	3 (7.5%)
Intermediate-risk	0.226–0.368	39	9 (23.1%)
High-risk	>0.368	39	21 (53.8%)

PNP, precancerous neoplastic polyps.

## 4 Discussion

We enrolled 589 patients who underwent hemorrhoid surgery at our center, of whom 167 had PNP, highlighting the need to identify high-risk patients in this cohort. The objective was to benchmark several machine learning approaches and identify the top-performing model. The RF model achieved the highest AUROC and exhibited the best overall performance; therefore, it was chosen as the final model.

In our study, age, sex, BMI, drinking, smoking, and hypertension were identified as factors associated with concomitant PNP among patients undergoing hemorrhoid surgery. In a study of 20,792 individuals aged ≥50 years, Corley et al. (2013) reported that the incidence of colorectal adenomas increased markedly with age and was substantially higher in men than in women. PNP show a similar pattern. Lieberman et al. (2014) reported that men were more likely to develop large polyps (>9 mm in diameter). In a cross-sectional study of 25,490 patients, Waldmann et al. (2016) reported a higher prevalence of PNP in men. A meta-analysis by Yam et al. (2025) including 12 studies showed that the overall prevalence of polyps was higher in older than in younger patients; notably, left-sided colonic polyps were more common in younger patients. Therefore, for older male patients, we recommend completing colonoscopy in routine clinical practice. In a meta-analysis including 43 studies, Bailie et al. (2017) reported that smoking and drinking were associated with a higher incidence of SP, with an even stronger association observed for sessile serrated adenoma/polyp. The same study also reported that higher BMI was associated with a higher prevalence of SP (Bailie et al., 2017). Burnett-Burnett-Hartman et al. (2011) reported that smoking was associated with a higher risk of adenomatous polyps. A case–control study (4,220 cases; 3,338 controls) showed that smoking, drinking, and higher BMI were linked to greater colorectal cancer risk (Carr et al., 2020). These observations are consistent with our results: among patients undergoing hemorrhoid surgery, PNP was more prevalent in those who reported drinking or smoking, or who had higher BMI. Hypertension may also be associated with colorectal cancer risk (O'Sullivan et al., 2022). Kaneko et al. (2021) observed that hypertension was linked to an elevated occurrence of colorectal neoplasms. Watanabe et al. (2015) conducted a cross-sectional analysis in 1,318 patients and found an independent association between antihypertensive medication use and colorectal polyps. Therefore, enhanced screening and evaluation may be warranted for patients with hypertension. In addition to risk prediction and early detection, proper management of patients at oncological risk is equally critical. A meta-analysis conducted by Chaouch et al. (2024) demonstrated that, among carefully selected patients with advanced rectal cancer, robot-assisted surgery represents a safe and feasible minimally invasive treatment option and may enhance specific perioperative outcomes.

PNP constitute an important premalignant stage in the development of colorectal cancer. In patients with hemorrhoids, manifestations such as hematochezia and perianal discomfort may be mistakenly attributed exclusively to the hemorrhoidal condition. Moreover, some patients may refuse colonoscopy because of the burden associated with repeated bowel preparation, concerns regarding repeated anesthesia, or financial limitations, thereby delaying the identification of colorectal lesions. Therefore, the early recognition of individuals at elevated risk for PNP among patients undergoing hemorrhoid surgery may reinforce recommendations for colonoscopy, promote the timely removal of precancerous lesions, and decrease the likelihood of missed early colorectal neoplasia. The pronounced risk gradient observed across probability-based strata indicates a substantially higher prevalence of PNP in the high-risk group (53.8%). In clinical practice, patients in the high-risk group should be strongly encouraged to undergo colonoscopy at the earliest opportunity. In contrast, patients in the low-risk group should not be regarded as exempt from evaluation, but should still adhere to routine colorectal cancer screening recommendations. Moreover, DCA suggests that the model may offer a net clinical benefit over default strategies, underscoring its practical utility. Therefore, accurately identifying high-risk patients based on available evidence remains a major challenge for clinicians. Our model identified factors associated with PNP in patients undergoing hemorrhoid surgery and ranked their relative importance. The clinical value of this model lies not in replacing colonoscopy, but in strengthening colonoscopic evaluation among patients undergoing hemorrhoid surgery. In this context, the present model serves not only as a tool for PNP prediction but also as a practical approach to the precision prevention of colorectal neoplasia in patients undergoing hemorrhoid surgery. It may also help patients better understand the rationale for the evaluation plan, thereby facilitating more efficient clinician–patient communication.

Several limitations warrant consideration. First, as a single-center retrospective study, all participants were recruited from the same hospital, which may restrict the generalizability of the results. Second, only patients who underwent hemorrhoid surgery and completed colonoscopy with pathological confirmation either before surgery or within 1 year postoperatively were included, which may introduce selection bias and temporal heterogeneity. Third, due to the retrospective design, only variables available in existing medical records were incorporated. Additional laboratory parameters and emerging molecular biomarkers may further enhance the predictive performance of the model (Deng et al., 2024b). Finally, although the RF model showed acceptable performance in the testing set, the decrease in AUROC from 0.892 in the training set to 0.738 in the testing set suggests potential overfitting. Therefore, multicenter prospective studies with independent external validation are needed to further assess the robustness and clinical applicability of the model.

## 5 Conclusion

This study integrated demographic characteristics, lifestyle-related variables, and histories of metabolism-related diseases among patients undergoing hemorrhoid surgery to establish a machine learning model with favorable predictive performance. The model offers robust data-driven support for clinical decision-making and facilitates the precise identification of individuals at elevated risk of PNP within this patient population. In addition, it may assist in recognizing those most likely to benefit from colonoscopy and in informing the formulation of individualized management strategies, thereby minimizing missed diagnoses and strengthening the early prevention of colorectal neoplasia.

## Data Availability

The raw data supporting the conclusions of this article will be made available by the authors, without undue reservation.
